# Low-Temperature UVO-Sintered ZnO/SnO_2_ as Robust Cathode Buffer Layer for Ternary Organic Solar Cells

**DOI:** 10.3390/nano12183149

**Published:** 2022-09-11

**Authors:** Zhijun Zou, Fen Li, Jing Fang, Mingxin Chen, Xiaoxiang Sun, Chang Li, Jiayou Tao, Gaohua Liao, Jianjun Zhang

**Affiliations:** 1 Key Laboratory of Hunan Province on Information Photonics and Freespace Optical Communications, College of Physics and Electronics, Hunan Institute of Science and Technology, Yueyang 414006, China; 2 College of Electronic Information and Optical Engineering, Nankai University, Tianjin 300071, China

**Keywords:** ternary organic solar cells, cathode buffer layer, ZnO nanoparticles, SnO_2_, UVO sintering

## Abstract

The cathode buffer layer (CBL) plays a crucial role in organic solar cells (OSCs), and it has been challenging to obtain high-quality CBL by using simple and reliable processes. In this paper, the bilayer structure consisting of ZnO nanoparticles (NPs) and sol–gel SnO_2_ was prepared by the low-temperature (<100 °C) UV-ozone (UVO) sintering process and used as the robust CBL for ternary OSCs based on PTB7-Th:PCDTBT:PC_70_BM. The results show that the insertion of SnO_2_ can effectively fill the cracks and pores on the surface of the ZnO NP film, thereby improving the overall compactness and flatness of the CBL and reducing the defect density inside the CBL. Furthermore, the insertion of SnO_2_ slightly improves the transmittance of the CBL to photons with wavelengths in the range of 400–600 nm, and also increases the electron mobility of the CBL thus facilitating the extraction and transport of the electrons. Compared to the devices using UVO-ZnO and UVO-SnO_2_ CBLs, the devices with UVO-ZnO/SnO_2_ CBL exhibit exceptional performance advantages, the best power conversion efficiency (*PCE*) reaches 10.56%. More importantly, the stability of the devices with ZnO/SnO_2_ CBL is significantly improved, the device (*PCE)* still maintains 60% of the initial value after 30 days in air. The positive results show that the UVO-ZnO/SnO_2_ is an ideal CBL for OSCs, and due to the low-temperature process, it has great application potential in flexible OSCs.

## 1. Introduction

Solar cells are of great significance in solving the energy shortage crisis and environmental pollution problems faced by mankind. Among various types of solar cells, organic solar cells (OSCs) have become a hot research topic in the field of solar cells due to the advantages of low cost, easy processing, flexible compatibility, and roll-to-roll large area production [1,2,3,4]. In recent years, the power conversion efficiency (*PCE*) of OSCs has increased substantially, and the laboratory *PCE* of the single-junction OSCs has now exceeded 19% [5,6]. The steady increase in *PCE* reveals the research value and application potential of OSCs.

It is well known that the cathode buffer layer (CBL) plays a crucial role in OSCs, and the deposition of the CBL is an important part of the OSCs preparation process, especially in the inverted OSCs, the device performance is largely dependent on the selection of the CBL material [7,8]. The widely used CBL material in inverted OSCs is metal oxide [9,10,11,12], while the zinc oxide (ZnO) nanoparticles (NPs) have become a more favored material by the researchers due to its higher electron mobility and photon transmission as well as lower work function [13,14,15,16]. Unfortunately, further improvement in the *PCE* of OSCs based on the ZnO NPs is hindered by the presence of defect states. The results of experimental and first-principle calculations have clarified the presence of defects in ZnO NPs [17,18,19]. The calculations based on density-functional theory (DFT) within the general gradient approximation plus Hubbard U indicates that the most favorable defects of the ZnO NPs are Zn and O vacancies [20]; the calculations based on exchange-correlation functionals demonstrates that the oxygen vacancies defect state appears in the valence band of ZnO and acts as a deep donor [21]. Undoubtedly, the photogenerated carriers (holes) annihilate once they meet the electrons released by the deep-level donors, resulting in the reduction of the short-circuit current density (*J_sc_*) and the deterioration of the devices performance. In addition, the ZnO NP films are likely to show surface cracks and pores during the deposition process [22,23,24], resulting in the organic active layer on top of it not being able to form close contact. In short, the higher defect density and poor surface morphology in the ZnO NPs CBL will inevitably increase the probability of the recombination of electrons and holes generated in the active layer [25].

Obviously, reducing the defect density of ZnO NPs CBL and improving the surface morphology quality are effective strategies to improve the *PCE* of OSCs. The interface engineering therefore has been carried out between the ZnO and active layer by inserting additional layers, such as conjugated polyelectrolytes, fullerene derivative, metal oxides and semiconductor NPs [26,27,28]. Previous studies by our group [29,30] have also shown that insertion layers such as water-soluble conjugated polymer PFN and germanium NPs can effectively reduce the surface defect density of the ZnO NP CBL and strengthen the interfacial contact between the active layer and the ZnO NPs CBL, thereby increasing the *J_sc_* and reducing the series resistance of the devices. Recently, the tin dioxide (SnO_2_) has appeared to be a better CBL for OSCs due to its high electron mobility, wide bandgap, and low defect density [31,32]. In fact, due to the excellent electrical properties, SnO_2_ has been widely used as CBLs for perovskite solar cells [33,34,35]. It is foreseeable that using ZnO/SnO_2_ as the CBL for OSCs, the synergistic effect of the two can effectively promote the *PCE* of OSCs.

In this paper, we used a facile two-step low-temperature (<100 °C) UV-ozone (UVO) sintering method to fabricate the ZnO/SnO_2_ bilayer as a robust CBL for the PTB7-Th:PCDTBT:PC_70_BM ternary OSCs. We observed that the introduction of SnO_2_ layer can significantly eliminate the cracks and pores on the surface of the ZnO NP film, making the surface morphology denser and smoother, thereby strengthening the interface contact between the CBL and the active layer. In addition, compared with the ZnO monolayer, the ZnO/SnO_2_ bilayer exhibits higher photon transmittance and electron mobility, which further improves the photon absorption of the active layer while optimizing the electrons transport and extraction. Benefiting from the adoption of the ZnO/SnO_2_ bilayer CBL, the optimum *PCE* of the devices was improved by 16.7%, reaching 10.56%. More importantly, the stability of the devices under air atmosphere was significantly enhanced. Our results show that the UVO-ZnO/SnO_2_ bilayer is an ideal CBL for OSCs, and due to the low-temperature sintering process, the ZnO/SnO_2_ bilayer has great application potential in flexible OSCs.

## 2. Experimental Details

### 2.1. Fabrication of ZnO/SnO_2_ Bilayer

**Figure 1:**
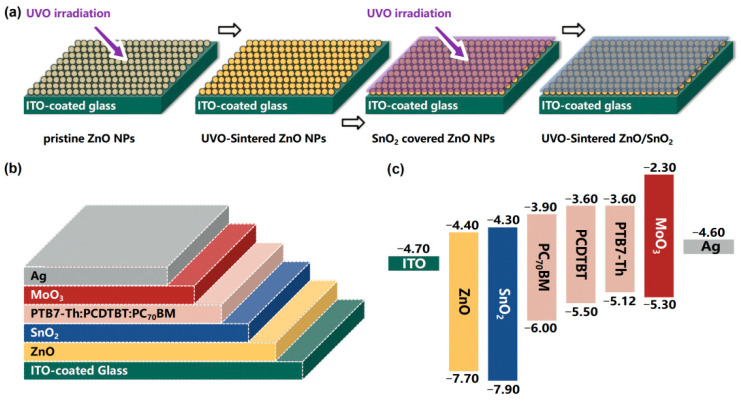
(**a**) The fabrication process of the two-step UVO-sintered ZnO/SnO_2_ bilayer. (**b**) The schematic architecture of the inverted ternary OSCs. (**c**) The energy level of the materials.

The fabrication process of the two-step UVO-ZnO/SnO_2_ bilayer is shown in Figure 1a. The preparation methods of water-bath ZnO NPs suspension and sol–gel SnO_2_ can be found in our previous work [35,36]. Generally, the ZnO NPs suspension was synthesized by hydrolysis and condensation of zinc acetate dihydrate by potassium hydroxide in methanol using a Zn^2+^:OH^−^ ratio of 1:1.7, and the sol–gel SnO_2_ was synthesized by dissolving tin dichloride dihydrate in ethanol to form a solution with a concentration of 0.1 M. The UVO cleaner (YZUV-22C, Beijing Kenuo Instrument Equipment Co., Ltd., Beijing, China) equipped with Hg lamp was used as the platform for irradiating the ZnO monolayer or ZnO/SnO_2_ bilayer film. The power density of the UV lamp is about 0.5 W/cm^2^ and the temperature of the substrates during UVO treatment is about 70 °C [29,35]. Firstly, the ZnO NPs was spin-coated on the cleaned commercial indium tin oxide (ITO) coated glass substrate (STN-SI-10, China Southern Glass Group Co., Ltd., Shenzhen, China) and subjected to UVO sintering, then the sol–gel SnO_2_ precursors was spin-coated on the UVO-ZnO NPs, and UVO sintering was performed again to finally obtain ZnO/SnO_2_ bilayer film. The sintering time of the ZnO-coated monolayer film and the ZnO/SnO_2_-coated bilayer film was 20 min and 60 min, respectively.

### 2.2. Fabrication of OSCs with ZnO/SnO_2_ CBL

The schematic architecture of the inverted ternary OSCs (ITO/ZnO NPs/SnO_2_/active layer/MoO_3_/Ag) are shown in Figure 1b. The mixed PTB7-Th:PCDTBT:PC_70_BM (0.8:0.2:1.5) was selected as the active layer materials; the mixing ratio optimization process has been described in detail elsewhere [37]. Before device fabrication, the ITO-coated glass substrate was cleaned by ultrasonic treatment in detergent, de-ionized water, acetone and isopropyl alcohol sequentially. Firstly, the ZnO/SnO_2_ bilayer CBL (about 100-nm thick) was prepared as described above. After, the substrate was transferred into a nitrogen filled glovebox, the blend active layer was deposited by spin-coating (2000 rpm for 30 s) from the pre-prepared PTB7-Th:PCDTBT:PC_70_BM blend solution, and the preparation method is as follows: the polymers (PTB7-Th and PCDTBT) and PC_70_BM (1:1.5 *w*/*w*) were co-dissolved in the mixed solvent of chlorobenzene and 1,8-diodooctane (97:3 vol/vol). The overall polymer concentration was 10 mg/mL and the solution was stirred at 90 °C for 12 h [37]. The thickness of the active layer is about 100 nm. Finally, a 10-nm thick molybdenum trioxide (MoO_3_) hole transport layer (HTL) and a 100-nm thick Ag electrode layer were subsequently evaporated through a shadow mask under the pressure of 7.0 × 10^−4^ Pa. The devices with the UVO-ZnO NPs CBL (about 50-nm thick) and the UVO-SnO_2_ CBL (about 50-nm thick) were also fabricated as reference.

### 2.3. Characterization

The current density–voltage (*J–V*) measurement of the ternary OSCs were conducted under simulated sunlight of 100 mW/cm^2^ using AM 1.5G type filter. The external quantum efficiency (EQE) spectra were tested using a Solar Cell Quantum Efficiency Measurement System (QEX10) from PV Measurement, Inc. Scanning electron microscopy (SEM) the atomic force microscopy (AFM) were used to investigate the morphology and roughness, the instrument models are JEOL JSM-7800F and Seiko instrumental SPA 400, respectively. Optical transmittance spectra were measured by spectrophotometer (Cary 5000 UV-VIS). The electron mobility in the electron-only devices was assessed using the space-charge-limited current (SCLC) method [38].

## 3. Results and Discussion

**Figure 2:**
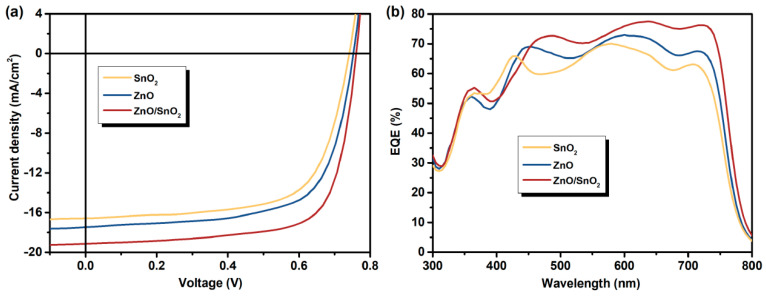
(**a**) The current density–voltage (*J–V*) characteristics and (**b**) the external quantum efficiency (*EQE*) curves of the devices with different CBLs.

The energy level of materials are illustrated in Figure 1c. Compared to ZnO, SnO_2_ exhibits a higher conduction band bottom and a lower valence band top. Apparently, for the ZnO/SnO_2_ bilayer CBL, the energy cascade of the conduction band bottom is more favorable for the electron extraction and transport [39], while the lower valence band top is more effective in blocking the holes. As a reference, the ternary OSCs based on UVO-ZnO NPs CBL and the UVO-SnO_2_ CBL were also prepared and fabricated. The current density–voltage (*J–V*) characteristics of the devices with UVO-ZnO, UVO-SnO_2_ and UVO-ZnO/SnO_2_ CBLs are shown in Figure 2a, and the corresponding detailed photovoltaic parameters are summarized in Table 1. The devices based on UVO-ZnO exhibits an optimum *PCE* of 9.05%, with open circuit voltage (*V_oc_*) of 0.761 V, *J_sc_* of 17.72 mA/cm^2^, and fill factor (*FF*) of 69.07%. When UVO-SnO_2_ is employed instead of UVO-ZnO as the CBL, the overall performance of the devices decreases, with a *J_sc_* of 16.33 mA/cm^2^, an *V_oc_* of 0.754 V, an *FF* of 67.97%, and an optimum *PCE* of only 8.37%. Compared to the devices using monolayer CBL, the devices with UVO-ZnO/SnO_2_ bilayer exhibit exceptional performance advantages. The best *PCE* of the devices reaches 10.56%, which is matched with a *J_sc_* of 19.03 mA/cm^2^, an *V_oc_* of 0.77 V, and an *FF* of 72.05%.

The positive effects of the UVO-ZnO/SnO_2_ bilayer CBL on the device performances can also be confirmed from the corresponding external quantum efficiency (EQE) characteristics as shown in Figure 2b. The devices with the UVO-ZnO/SnO_2_ bilayer CBL exhibits a prominent enhancement response in the wavelength range of 450–750 nm as compared to that of the devices with UVO-ZnO and UVO-SnO_2_ CBLs, resulting in a substantial increase in *J_sc_*. In addition, the EQE value of the devices with UVO-ZnO/SnO_2_ CBL surpasses 70% at around 600–700 nm, indicating an efficient photo-to-electron conversion.

**Figure 3:**
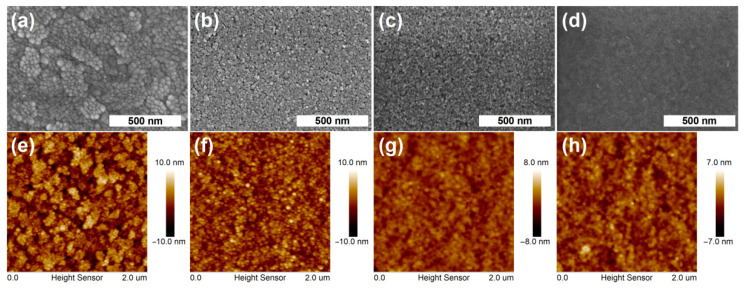
The SEM (**a**–**d**) and AFM (**e**–**h**) images of different films, (**a**,**e**) ITO, (**b**,**f**) UVO-ZnO, (**c**,**g**) UVO-SnO_2_, (**d**,**h**) UVO-ZnO/SnO_2_.

To further explore the positive effects of the UVO-ZnO/SnO_2_ bilayer CBL on the device performances, the surface morphology of CBLs are observed by using the SEM and AFM, and the results are shown in Figure 3, the surface morphology of the ITO are also given for reference. Figure 3a–d exhibits the SEM images for the different films. As mentioned earlier, the ZnO NP film are prone to surface cracks and pores, as in the case revealed in Figure 3b, which will undoubtedly hinder the intimate contact between the active layer and the CBL and thus increase the interfacial contact resistance. The surface cracks and pores also exist in the UVO-SnO_2_ film, as displayed in Figure 3c, but the density and scale of the cracks and pores are relieved, which makes the surface of the SnO_2_ film denser and smoother. By depositing UVO-SnO_2_ on top of the ZnO NP film, the cracks and pores on the surface of the SnO_2_ film basically disappeared, and the surface showed a more dense and smooth morphology, as shown in Figure 3d. The flat and smooth surface morphology of SnO_2_ films also implies that the cracks and pores on the surface of the ZnO NP film are effectively covered and filled [40]. The results obtained by the AFM are consistent with the SEM, Figure 3e–h display the AFM images for different CBLs. In Figure 3f, the ZnO NP film exhibits a root-mean-square (RMS) roughness of 3.19 nm at a scan scale of 2 × 2 um. The presence of surface defects on the particle-featured morphology increased the possibility of the excitons trapping and recombination. When the ZnO NP film was covered by SnO_2_, the surface became smoother with a RMS roughness of 1.00 nm (as shown in Figure 3h), indicating that the cracks and pores between the ZnO NPs are effectively filled by SnO_2_ and the film is planarized. The reduced roughness of the ZnO/SnO_2_ bilayer film shows fewer traps, which contribute to increasing *J_sc_* and *FF* [7].

**Figure 4:**
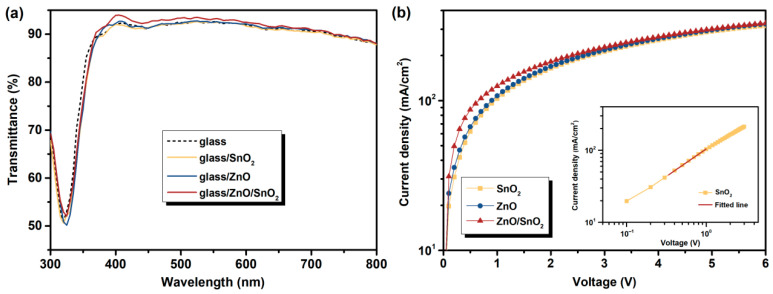
(**a**) The optical transmittance spectra of the different CBLs on the glass substrate. (**b**) The *J-V* characteristics of the electron-only devices with the configuration of ITO/CBLs/ternary active layer/Al. The inset is the calculation method of the electron mobility.

The insertion of the SnO_2_ layer not only improves the interface contact between the active layer and the CBL, but also optimizes the optical and electrical properties of the CBL. Figure 4a presents the optical transmittance spectra of the UVO-ZnO, UVO-SnO_2_ and UVO-ZnO/SnO_2_ CBLs. The results show that the additional inserted SnO_2_ layer does not negatively affect the optical properties of the ZnO layer, but slightly improves the transmittance to the photons with wavelengths in the range of 400–600 nm. This interesting phenomenon has also been reported in other literature [30,35], and one of the possible reasons should be that the smooth surface is more conducive to the transmission of photons [41]. In this case, the UVO-ZnO/SnO_2_ shows a smoother surface compared with the UVO-ZnO and UVO-SnO_2_, as revealed by the AFM images. More photons pass through the CBL into the active layer, which enhances the photon absorption in the active layer and helps to improve the *J_sc_* of the devices. Moreover, the electrical properties of the CBL is also critical to the *PCE* of the devices. The electron transfer characteristics of the different CBLs are investigated by using the space-charge-limited current (SCLC) model, and the electron-only devices (ITO/CBLs/ternary active layer/Al) are fabricated. The electric field-dependent electron mobilities in the active layer were calculated through the following equation $J=(9/8)\varepsilon_{0}\varepsilon_{r}\mu ((V^{2})/(L^{3}))$ [38], where ε_0_ is the permittivity of free space, ε_r_ is the relative permittivity of the material, μ is the hole (μ_h_) or electron (μ_e_) mobility, V is the applied voltage and L is the thickness of the active layer. As shown in Figure 4b, the UVO-ZnO/SnO_2_ devices exhibit the highest electron current density, which will contribute to the enhanced *J_sc_* for the corresponding devices. The electron mobility was calculated by fitting the space charge limited region according to Childʹs law (as displayed in the inset in Figure 4b), and the calculated electron mobility is 4.3 × 10^−4^ cm^2^V^−1^s^−1^ for the UVO-ZnO, 4.6 × 10^−4^ cm^2^V^−1^s^−1^ for the UVO-SnO_2_ and 8.1 × 10^−4^ cm^2^V^−1^s^−1^ for the UVO-ZnO/SnO_2_. The devices with UVO-ZnO/SnO_2_ exhibit the highest electron mobility, which will undoubtedly achieve the most efficient electron extraction and transport, thereby improving the *J_sc_* and *FF* of the devices.

**Figure 5:**
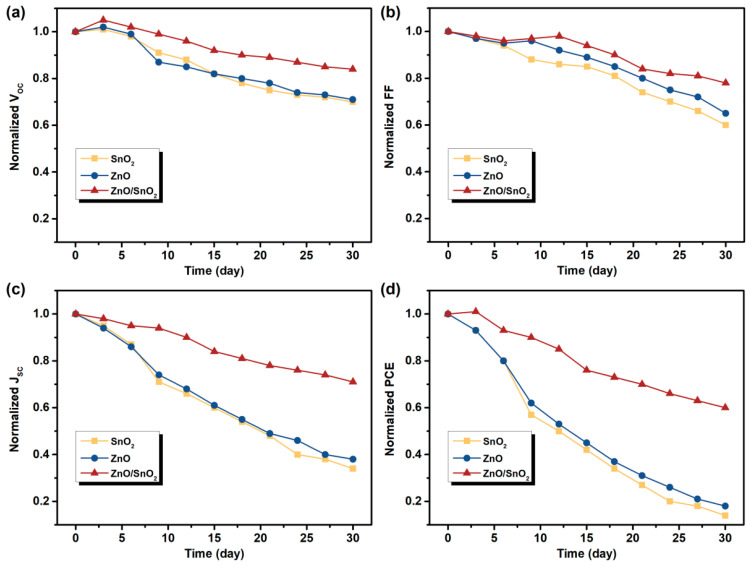
Stability measurements in ambient air. The variation of normalized (**a**) *V_oc_*, (**b**) *FF*, (**c**) *J_sc_* and (**d**) *PCE* over a period of 30 days for the devices with UVO-ZnO, UVO-SnO_2_ and UVO-ZnO/SnO_2_ CBLs.

Finally, the long-term stability of OSCs represents a crucial factor for the commercialization. Therefore, it is necessary to clarify the effect of the UVO-ZnO/SnO_2_ bilayer CBL on device lifetime. Hereupon, the degradation of the photovoltaic parameters (*V_oc_*, *FF*, *J_sc_* and *PCE*) of the devices with different CBLs for a period of 30 days is evaluated. Figure 5 show the variation of the normalized values of those photovoltaic parameters over the aging period. In the devices with UVO-ZnO and UVO-SnO_2_ CBLs, a strong degradation of all the photovoltaic parameters are observed. The devices lost more than 80% of its initial *PCE* after 30 days. On the contrary, in the devices with UVO-ZnO/SnO_2_ CBL, the *V_oc_* and *FF* preserve about 60% of the initial values after 30 days. Although the attenuation of *J_sc_* is more obvious compared to the *V_oc_* and *FF*, the *PCE* device still maintains 60% of the initial value. The use of UVO-ZnO/SnO_2_ CBL significantly improves the device lifetime, an expected result that can be attributed to the following reasons: on the one hand, SnO_2_ effectively fills the cracks and pores on the surface of the ZnO NP film, making the CBL more dense and acting as a barrier to H_2_O and O_2_ molecules in air; on the other hand, the UVO sintering process itself passivates defects in the ZnO NP film, as confirmed by our previous work [29], and it is well established that oxygen chemisorption occurs readily onto interstitial zinc sites present on the oxide surface [42]. The enhanced denseness and the reduced defect density of the CBL have contributed to the significant improvement in device stability.

## 4. Conclusions

In summary, the bilayer structure consisting of ZnO NPs and sol–gel SnO_2_ was prepared by the low-temperature (<100 °C) UVO sintering process and used as the CBL for ternary OSCs based on PTB7-Th:PCDTBT:PC_70_BM. The results show that the insertion of SnO_2_ can effectively fill the cracks and pores on the surface of the ZnO NP film, thereby improving the overall compactness and flatness of the CBL and reducing the defect density inside the CBL. Furthermore, the additional inserted SnO_2_ does not negatively affect the optical properties of the ZnO, but slightly improves the transmittance to the photons with wavelengths in the range of 400–600 nm, and also increases the electron mobility of the CBL, thereby facilitating the extraction and transport of the electrons. Compared to the devices using UVO-ZnO and UVO-SnO_2_ CBLs, the devices with UVO-ZnO/SnO_2_ CBL exhibit exceptional performance advantages, the best *PCE* of the devices reaches 10.56%, which is matched with a *J_sc_* of 19.03 mA/cm^2^, an *V_oc_* of 0.77 V, and an *FF* of 72.05%. More importantly, the stability of the devices with ZnO/SnO_2_ CBL is significantly improved due to the enhanced denseness and reduced defect density of the CBL, the *PCE* device still maintains 60% of the initial value after 30 days in air. Our positive results show that the UVO-ZnO/SnO_2_ bilayer is an ideal CBL for OSCs, and due to the low-temperature process, the ZnO/SnO_2_ bilayer has great application potential in flexible OSCs.

## Figures and Tables

**Table 1:** The photovoltaic parameters for the devices with different CBLs. The *PCE* parameters give the mean and error.

CBLs	*J_sc_* (mA/cm^2^)	*V_oc_* (V)	*FF* (%)	*PCE* (%)
UVO-ZnO	17.72	0.761	69.07	9.05 (8.45 ± 0.27)
UVO-SnO_2_	16.33	0.754	67.97	8.37 (7.78 ± 0.28)
UVO-ZnO/SnO_2_	19.03	0.770	72.05	10.56 (9.94 ± 0.32)

## Data Availability

The article contains all the data.
